# High-order brain interactions in ketamine during rest and task: a double-blinded cross-over design using portable EEG on male participants

**DOI:** 10.1038/s41398-024-03029-0

**Published:** 2024-07-27

**Authors:** Rubén Herzog, Florentine Marie Barbey, Md Nurul Islam, Laura Rueda-Delgado, Hugh Nolan, Pavel Prado, Marina Krylova, Igor Izyurov, Nooshin Javaheripour, Lena Vera Danyeli, Zümrüt Duygu Sen, Martin Walter, Patricio O’Donnell, Derek L. Buhl, Brian Murphy, Agustin Ibanez

**Affiliations:** 1 Latin American Brain Health Institute, Universidad Adolfo Ibañez, Santiago de Chile, Chile; 2 Sorbonne Université, Institut du Cerveau - Paris Brain Institute - ICM, Inserm, CNRS, Paris, France; 3 Cumulus Neuroscience Ltd, Dublin, Ireland; 4 Escuela de Fonoaudiología, Facultad de Odontología y Ciencias de la Rehabilitación, Universidad San Sebastián, Santiago, Chile; 5 Department of Psychiatry and Psychotherapy, Jena University Hospital, Jena, Germany; 6 German Center for Mental Health (DZPG), partner site Halle-Jena-Magdeburg, Jena, Germany; 7 Neuroscience Drug Discovery Unit, Takeda Pharmaceuticals, Cambridge, MA 02390 USA; 8 Global Brain Health Institute, UCSF and Trinity College Dublin, Dublin, Ireland

**Keywords:** Pharmacology, Neuroscience

## Abstract

Ketamine is a dissociative anesthetic that induces a shift in global consciousness states and related brain dynamics. Portable low-density EEG systems could be used to monitor these effects. However, previous evidence is almost null and lacks adequate methods to address global dynamics with a small number of electrodes. This study delves into brain high-order interactions (HOI) to explore the effects of ketamine using portable EEG. In a double-blinded cross-over design, 30 male adults (mean age = 25.57, SD = 3.74) were administered racemic ketamine and compared against saline infusion as a control. Both task-driven (auditory oddball paradigm) and resting-state EEG were recorded. HOI were computed using advanced multivariate information theory tools, allowing us to quantify nonlinear statistical dependencies between all possible electrode combinations. Ketamine induced an increase in redundancy in brain dynamics (copies of the same information that can be retrieved from 3 or more electrodes), most significantly in the alpha frequency band. Redundancy was more evident during resting state, associated with a shift in conscious states towards more dissociative tendencies. Furthermore, in the task-driven context (auditory oddball), the impact of ketamine on redundancy was more significant for predictable (standard stimuli) compared to deviant ones. Finally, associations were observed between ketamine’s HOI and experiences of derealization. Ketamine appears to increase redundancy and HOI across psychometric measures, suggesting these effects are correlated with alterations in consciousness towards dissociation. In comparisons with event-related potential (ERP) or standard functional connectivity metrics, HOI represent an innovative method to combine all signal spatial interactions obtained from low-density dry EEG in drug interventions, as it is the only approach that exploits all possible combinations between electrodes. This research emphasizes the potential of complexity measures coupled with portable EEG devices in monitoring shifts in consciousness, especially when paired with low-density configurations, paving the way for better understanding and monitoring of pharmacological-induced changes.

## Introduction

Ketamine, a non-competitive N-Methyl-d-aspartate (NMDA) receptor antagonist considered a non-serotonergic psychedelic compound, has garnered attention for its capacity to induce alterations in the global dynamics of conscious states [1, 2]. It holds promising implications for pharmacological interventions, especially in the treatment of depression and other mood-related disorders [3, 4]. Specifically, it has been linked to experiences of derealization (i.e., feeling detached from surroundings), depersonalization (i.e., feeling detached from self), and altered perception of the body, environment, and time [1, 2]. Various studies employing behavioral analyses and neuroimaging have illustrated shifts in neural patterns during ketamine administration (see reviews [5, 6]). Although ketamine does not directly target serotonergic receptors like classical psychedelics, evidence from neuroimaging and electrophysiological studies suggest common signatures and potential mechanisms of altered states of consciousness [7–9]. Both ketamine and serotonergic psychedelics have been linked to modulations of the Default Mode Network (DMN) [10] as well as increases of brain entropy [7, 11]. Many of the brain pattern shifts observed under ketamine and psychedelics entail a decrease in top-down brain organization paired with an enhanced emphasis on the lower hierarchies of sensory information [12–15]. These observations are consistent with the Relaxed Beliefs Under Psychedelics (REBUS) model proposed by Carhart-Harris and Friston, which suggests that psychedelics alter consciousness by reducing the weight of prior beliefs when processing bottom-up sensory information emerging from the periphery [15]. The reduced top-down control from higher brain hierarchies results in a richer conscious experience as well as sometimes prediction errors manifesting as perceptual illusions or experience of dissociation [12, 15]. During the ketamine experience, this dissociation may be further evidenced through complexity measures of brain activity, as reflected in increased entropy [15–18].

Emergent innovations in clinical trials emphasize the adoption of portable dry electrodes, addressing a significant gap in the demand for accessible, reliable, and economical biomarkers to monitor drug effects [19, 20]. However, studies exploring the effects of ketamine with low-density electrodes are scarce. An innovative and robust approach to probe into these effects in low-density setups encompasses brain high-order interactions (HOI) [21]. Three salient features made HOI critically relevant [22–26]. First, as opposed to standard event-related potentials (ERP), oscillations, and connectivity metrics, HOI can compute all possible interactions between signals (here, electrodes). Despite the advantages of having high-density arrays (as source localization, increased spatial resolution, interpolation of bad electrodes), the HOI approach becomes computationally challenging in these arrays due to the so-called combinatorial explosion, inducing a selection bias imposed by optimization techniques, such as greedy search [25], random sampling or simulated annealing [27]. In contrast, despite their reduced spatial resolution, low-density arrays allow us to feasibly compute all the possible interactions, thereby maximizing information conveyance—a feature unmatched by any other technique—and avoiding the potential bias introduced by optimization algorithms. Thus, leveraging HOI provides a unique and novel improvement to understanding brain dynamics using low-density arrays, which usually reduce data’s granularity. Second, HOI effectively capture the global dynamics of brain organization [22–26], a critical component of different consciousness states (disorder of consciousness, anesthesia, transitions and conscious access [28–32]), making them ideal for elucidating the effects of pharmacology. Furthermore, the evidence underscores HOI’ superior robustness when compared to traditional connectivity metrics across various modalities, including fMRI and EEG, and across different brain conditions [23–26]. In brief, when combined with entropy measures and low-density EEG, HOI may constitute a crucial approach for assessing global dynamic changes induced by ketamine and other drugs used in clinical trials settings.

**Fig. 1:**
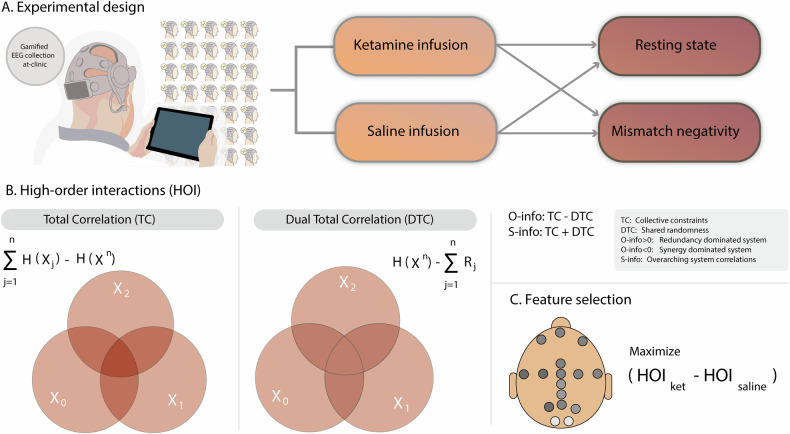
Overview of experimental design and data analysis. **A** Subjects participated in a double-blind crossover design using portable EEG, capturing both resting states and task-based recordings (namely, a gamified oddball paradigm inducing a typical mismatch negativity). In randomized sessions, participants received both ketamine and saline infusions. **B** Analysis of high-order interactions (HOI) entailed measurements of total correlation (TC) and dual total correlation (DTC), O-information and S-information (see section “Methods”). **C** Feature selection across the two designs (rest and task) was employed to pinpoint the primary differences between the ketamine and saline conditions.

The current study (Fig. 1) investigated changes in HOI during drug-induced altered state of consciousness, adopting a double-blinded cross-over design, enrolling 30 adults (but only 29 were used in this analysis), and administering racemic ketamine via a continuous infusion protocol (Fig. 1A). We juxtapose the effects of ketamine against a saline infusion as a control, leveraging both task-driven (gamified auditory oddball paradigm) and resting-state EEG sampling methodologies [33, 34]. Using a low-density, wireless, dry electrode EEG system [35], participants’ responses to ketamine were captured. Alongside EEG data, the subjective effects of ketamine administration were measured through self-reported and clinician-administered questionnaires. Our analyses focused on HOI assessed with entropy-based measures [36]. We predicted that HOI will yield consistent results across designs (resting state, task) and conditions (ketamine vs saline, and deviant vs. standard stimuli in the task). We expected that effects would mainly be observed in an alpha band which is systematically reported to be altered in ketamine studies [33, 34]. Such effects are anticipated to be more pronounced during resting state than in task [37], given ketamine’s hypothesized role in diminishing top-down control [15]. This reduced inhibitory control over sensory areas could manifest as an increase in redundancy, as sensory signals could now be amplified and propagated to more brain regions. To assess this specific hypothesis of increased redundancy associated with reduction of top-down hierarchical control, we include other measures of global dynamics (see below). Furthermore, during task-driven conditions, particularly a passive and gamified auditory oddball paradigm, the more predictable stimuli (i.e., standard) are expected to yield similar increased effects in contrast to the deviant stimuli, as prediction may be more impacted by the decreased top-down influences. Finally, we assessed whether HOI-associated changes during ketamine administration correlate with subjective consciousness altered states in terms of dissociative states (i.e., derealization scores) [38–40]. In brief, this study aims to establish the ability of a portable, low-density dry EEG device to capture the overarching global brain dynamics of ketamine-induced shifts, both in resting and task-engaged states, serving as potential brain signatures of altered states of consciousness.

## Methods

EEG data (16 electrodes, Cumulus Neuroscience dry-sensor 16 electrode headset) were recorded during a resting state and a mismatch negativity task under saline or ketamine infusion (Fig. 1A). A pipeline based on multivariate information theory was applied to investigate whether saline could be discriminated against ketamine under both rest and task. The complete set of possible combinations between electrodes at all orders of interactions (from 2 to 16) was assessed (Fig. 1B, C). The EEG used here has been presented previously on reference [41], where a detailed description can be found.

### Participants

Study participants (N = 30 males, 25.57 ± 3.74 y) were carefully selected to ensure a consistent and controlled environment for the research. To qualify, individuals had to be males aged between 18 and 55. Women were excluded to avoid any risk of undetected pregnancy and to reduce sample variability. Participants’ health was critically assessed through a physical examination, medical history, vital signs, a 12-lead Electrocardiogram (ECG), and clinical laboratory tests. Participants were excluded if they had a current or past history of psychiatric disorders as per the ICD-10, especially those with a history of drug or alcohol dependence/abuse in the last 6 months. Furthermore, any serious unstable illnesses, including but not limited to hepatic, renal, respiratory, cardiovascular, endocrine, and neurological disorders, led to exclusion. This also encompassed subjects with unresolved seizure causes, conditions that likely alter brain morphology or physiology such as uncontrolled hypertension or diabetes, significant acute illnesses a week prior to the drug administration, notable history of drug or food allergies, and the use of specific medications including antidepressants, anti-psychotics, anxiolytics, and others. Additionally, color-blind individuals were also not considered for this study. Each participant’s commitment to the study’s guidelines and restrictions was crucial, and they had to demonstrate their understanding and willingness to participate.

Ethics for this study was approved by the Institutional Review Board of the Otto-von-Guericke University Magdeburg, Germany, in accordance with the Declaration of Helsinki, and informed consent was obtained from all participants (Approbation code number: 123/18).

### Study design

This study was a placebo-controlled, double-blind, randomized, cross-over study designed to investigate the acute and persistent effects of ketamine on EEG and behavioral measures.

Participants were invited to the laboratory on five different occasions to complete repeated measurements: at enrolment/screening (visit 1); on the days of infusion of ketamine or saline placebo (visits 2 and 4); and on the days after infusion (visits 3 and 5). The two infusion days took place four weeks apart following the same study protocol, while timing of ketamine or saline administration was counterbalanced (Fig. 1). Throughout the study, additional task-driven EEG data collection was remotely performed by participants unsupervised in the home, for a week period before and after each infusion session (not analyzed here). All EEG recordings were performed with the portable dry EEG system developed by Cumulus Neuroscience (www.cumulusneuro.com). The analyses presented in this manuscript correspond to the data collected during the ketamine and saline infusions. The whole experiment was performed just once in the same laboratory, so no replication has been made. The power calculation for this study has an 80% chance of detecting a treatment difference at a significance level of 0.05 (two-sided), assuming the true difference between treatments is 0.750 units, assuming a within-patient standard deviation of the response equalt to 1.

### Ketamine infusion

During the infusion, participants were seated in a comfortable chair and their legs were elevated on a footrest. Overall mobility was restricted as a cannula was placed in each arm; one for the delivery of ketamine or saline, and the other to draw blood samples. Participants were administered a single IV infusion (of a total volume of 50 ml) of 0.5 mg/kg of racemic ketamine hydrochloride, over 40 mins, or IV saline solution (0.9%) over 40 min. During the infusions, the tablet was mounted on a tablet holder and operated by the researchers as participants were not able to bend their arms at that time.

### EEG recordings

EEG data was collected using the wireless 16-channel dry sensor EEG system developed by Cumulus Neuroscience (Cumulus, www.cumulusneuro.com), suitable for use in a variety of supervised and unsupervised settings [35]. The analog front-end is based on the ADS1299 chip-set from Texas Instruments, incorporating high input impedance of 1GΩ, a configurable driven bias function for common-mode rejection, built-in impedance checking, and configurable gain and sampling rates. The left mastoid is used for reference and the right mastoid for driven-bias, with single-use, snap-on electrodes attached to wires extending from the headset. An onboard processor and Bluetooth module transmit 250 Hz EEG data to another device (an Android tablet in this case), transferring it to a cloud server for storage and processing. Flexible Ag/AgCl coated polymer sensors of a comb-design (ANT-Neuro/eemagine GmbH) are used to achieve a stable and dermatologically safe contact to the scalp through the hair. The electronics and sensors are mounted on a flexible neoprene net for comfort and ease of montage. The incorporation of natural landmarks in the headset form factor and the stretchable structure both enable consistent placement by non-experts in line with the 10–20 sensor system.

### Resting-state session

Eyes-closed resting state EEG recordings were collected during the first 20 min of the 40 min continuous infusions. We expected ketamine effects to build-up linearly over time as the plasma concentration increased. For this reason and to maximize detection of any effects, we analyzed the later half of the resting state data during which ketamine concentration would be higher. Participants were instructed to rest with their eyes closed and remain still during the recording.

### Passive-listening auditory oddball paradigm

During the last 15 min of each infusion, we used an app-based version of the passive auditory oddball task developed by Cumulus— Sonic Scenes—eliciting the Mismatch Negativity (MMN) of infrequent ‘deviant’ stimuli in a train of ‘standard’ stimuli. The task was performed passively – the subject merely needed to remain still while listening to repetitive auditory stimuli. The participant was prompted to put on headphones and adjust the volume until he could clearly hear some sample tones. When ready, the participant asked to press play on a silent movie, which lasted 15 min. Tones played throughout, while the participant watched the film. Eight short films were used in an arbitrary cycled order, each consisting of silent clip montages compiled from stock footage. There were no narratives nor subtitles. Each movie session incorporated 1000 ‘standard’ tones (100 ms; 1000 Hz) and 200 pitch deviants (100 ms; 1200 Hz). The inter-onset interval was of 650 ms fixed as recommended by Duncan et al. [42]. To verify that the sound volume selected by participants for the gamified MMN task was stable throughout the study, we ran a linear regression on the recorded tablet volume against the session number, i.e. the number of times a user has performed the MMN task. Neither initial volume nor final volume - i.e. volume after incorporating any volume changes within the task - demonstrated an increase with session number (See Supplementary Table 3).

### Subjective scores of dissociative states

We assessed the Clinician-Administered Dissociative States Scale (CADSS), which measures dissociative states [43], and the 5D-ASC, which assesses self-reported altered states of consciousness [44]. CADSS has 3 subscales (“Amnesia”, “Derealization”, “Depersonalization”), while 5D-ASC entails five dimensions (“Oceanic Boundlessness”, “Dread of Ego Dissolution (DED)”, “Visionary Restructuralization”, “Auditory Alterations”, “Vigilance Reduction“). Both questionnaires were administered in the laboratory 1 h before and 1.5 h after the infusion. When answering after the infusions, participants were asked to answer according to the highest point of dissociative experience during infusion. The scores’ difference between before and after infusion were used to rate the subjective changes both for saline and for ketamine infusion. All questionnaires used validated German-language versions, except for the CADSS questionnaire which was translated by the research team as no validated translation was available at the time the study was conducted.

### EEG data preprocessing

In the Cumulus platform, EEG data is automatically uploaded to the cloud and a proprietary processing pipeline is applied, which is designed to quantify signal quality, and selectively aggregate individual trials to deal with the noise and signal variation seen in less controlled dry-sensor recordings. This verifies the integrity of timing information and excludes bad quality signal portions. Corrective procedures are applied for missing and anomalous data, including eye and other characteristic artifacts. After that, EEG signals were pre- processed with filtering from 0.5–40 Hz, epoch extraction, and baseline adjustment. All data were recorded with a left-mastoid reference.

To preserve the same number of channels (16) during the whole analysis, time points from all channels were removed if at least one channel had a flat signal in those points, which was required to explore all the possible high-order interactions. One subject was removed from further resting state analysis because there was <1 s of valid data in the ketamine condition. No differences in the number of selected points were found after the artifact removal (Supplementary Tables 1 and 2) across conditions (ketamine/saline) and recordings (rest/task). Resting state data were bandpass filtered in the canonical EEG frequency bands: δ: 0.5–4 Hz, θ: 4–8 Hz, α: 8–12 Hz, β: 12–30 Hz, γ: 30–40 Hz). Note that the definition of the γ range here has been elsewhere used for EEG [45] and ketamine [46] and should not be confused with the γ range used in intracranial recordings, which reaches much higher frequencies. Given that our goal was not to compute ERPs, we pooled all the valid trials (with at least 125 ms valid signal) corresponding to the deviant and standard tones separately to perform the analysis. In the following analysis, the 5 bands plus broadband (0.5–40 Hz) data was used for the resting state, while for the tasks only broadband data were used to focus only on the evoked rather than in the induced response. EEG data obtained appeared reliable as the standard effects on ketamine vs. saline solution were noticeable in the EEG spectrum (across the alpha peak and typical frequency/power decay, see Fig. S1).

### Pairwise and high-order interactions

To assess the hypothesis of ketamine-induced specific effects of redundancy in brain dynamics, different measures of entropy characterizing different properties of brain dynamics were computed. We used tools from multivariate information theory i) to quantify the nonlinear statistical dependencies between all the possible combinations between electrodes and ii) to distinguish the nature of these dependencies in terms of collective constraints (total correlation, TC), shared randomness (dual total correlation, DTC), synergy (O-info<0), redundancy (O-info>0), and overarching correlations (S-info) (see reference [36] for a detailed explanation of these measures).

Consider a system of *n* random variables denoted by X^n^ = (X_1_, …, X_n_). The TC, DTC, O-information (O) and S-information (S) are generalizations of the mutual information (MI) [36], which can be respectively expressed as:

1 $${TC}({X}^{n})=\mathop{\sum }\limits_{i=1}^{n}H({X}_{i})-H({X}_{1},...,{X}_{n})$$

2 $${DTC}({X}^{n})=H({X}_{1},...,{X}_{n})-\mathop{\sum }\limits_{i=1}^{n}H({X}_{i}{\rm{|}}{{X}^{n}}_{-i})$$

3 $$O({X}^{n})={TC}({X}^{n})-{DTC}({X}^{n})$$

4 $$S({X}^{n})={TC}({X}^{n}+{DTC}({X}^{n})$$

where $H({X}_{1},...,{X}_{n})$ is the joint Shannon’s entropy of the n variables, $H({X}_{i})$ the entropy of the i-th region and $H({X}_{i}|{{X}^{n}}_{-i})$ is the entropy of the i-th region conditioned by the activity of the whole system without it - which is known as “residual entropy,” and is denoted as ${R}_{i}$. Above, X_-i_^n^ stands for the vector of all variables except X_i_, i.e., (X_1_, …, X_i-1_, X_i+1_, …, X_n_). Estimations were performed using the Gaussian copula approximation [25, 47]. As for n = 2 TC = DTC = mutual information, only the TC was computed for each possible pair of electrodes. For the high-order interactions (from 3 to 16) all metrics were computed for each possible combination of electrodes at each order of interaction (Fig. 1B, C). Despite the mutual information is directly related to the Pearson correlation coefficient in the pairwise case, the Gaussian copula approach, by preserving the copula function between the variables, can capture nonlinear high-order dependencies as sinergy [48]. Although pairwise interactions completely define a multivariate Gaussian, entropy computation makes use of the determinant of the matrix, which is a property of the whole matrix rather than of the pairwise interactions (for a notable example of high-order statistical interactions in a full pairwise system see reference [49]).

Broadband (0.5–40 Hz) and filtered EEG signals (δ: 0.5–4 Hz, θ: 4–8 Hz, α: 8–12 Hz, β: 12–30 Hz, γ: 30–40 Hz) were analyzed considering all the possible combinations of electrodes at each order of interaction, here denoted by **k** (120 interactions for **k** = 2, 560 for **k** = 3, 1.820 for **k** = 4, 4.368 for **k** = 5, 8.008 for **k** = 6, 11.440 for **k** = 7, 12.870 for **k** = 8, 11.440 for **k** = 9, 8.008 for **k** = 10, 4.368 for **k** = 11, 1.820 for **k** = 12, 560 for **k** = 13, 120 for **k** = 14, 16 for **k** = 15 and 1 for **k** = 16). An *n-plet* represents a particular combination of *n* electrodes, and the effect of ketamine on it was assessed via the effect sizes.

### Effect sizes

To characterize the size of the effect associated with each measurement we used the Cohen’s $d$ effect size for paired samples [50]:

5 $$d=\frac{{\mu }_{{ket}}-{\mu }_{{sal}}}{s}$$

where ${\mu }_{{ket}}$ and ${\mu }_{{sal}}$ are the average measure of the ketamine and saline condition, respectively, and $s$ is the standard deviation of the difference of means (i.e ${\mu }_{{ket}}-{\mu }_{{sal}}$). This metric measures a standardized mean difference between paired samples, and its sign indicates the direction of the effect, i.e. if d > 0 means that ketamine increases the measure.

### Statistical analyses

For the selected features, a two-sided non-parametric Wilcoxon sign rank test for paired samples with the False Discovery Rate (FDR) correction for multiple comparisons was performed. As in previous work on HOI [22–26], statistical correction was not directly assessed for each HOI given the non-selective data approach including all interactions [51]. Conversely, we used Cohen’s D to report the effect size of HOI [22, 24, 25] as *p*-values can be artificially inflated. To compute the association between HOI and subjective scores, the change (ketamine - saline) in HOI was (Pearson) correlated with the change in subjective scores, yielding one R2 per n-plet and subjective score. We evaluated the significance of associations by a permutation test followed by post-hoc FDR correction for multiple comparisons. First, we generated a null distribution of R2 values by randomly permuting the correspondence between HOI and subjective scores 1000 times. Only correlations values above the 99.9-th percentile of their respective null distribution were then submitted to FDR correction (p < 0.005).

## Results

### Ketamine increases redundancy in the alpha band during resting state

**Fig. 2:**
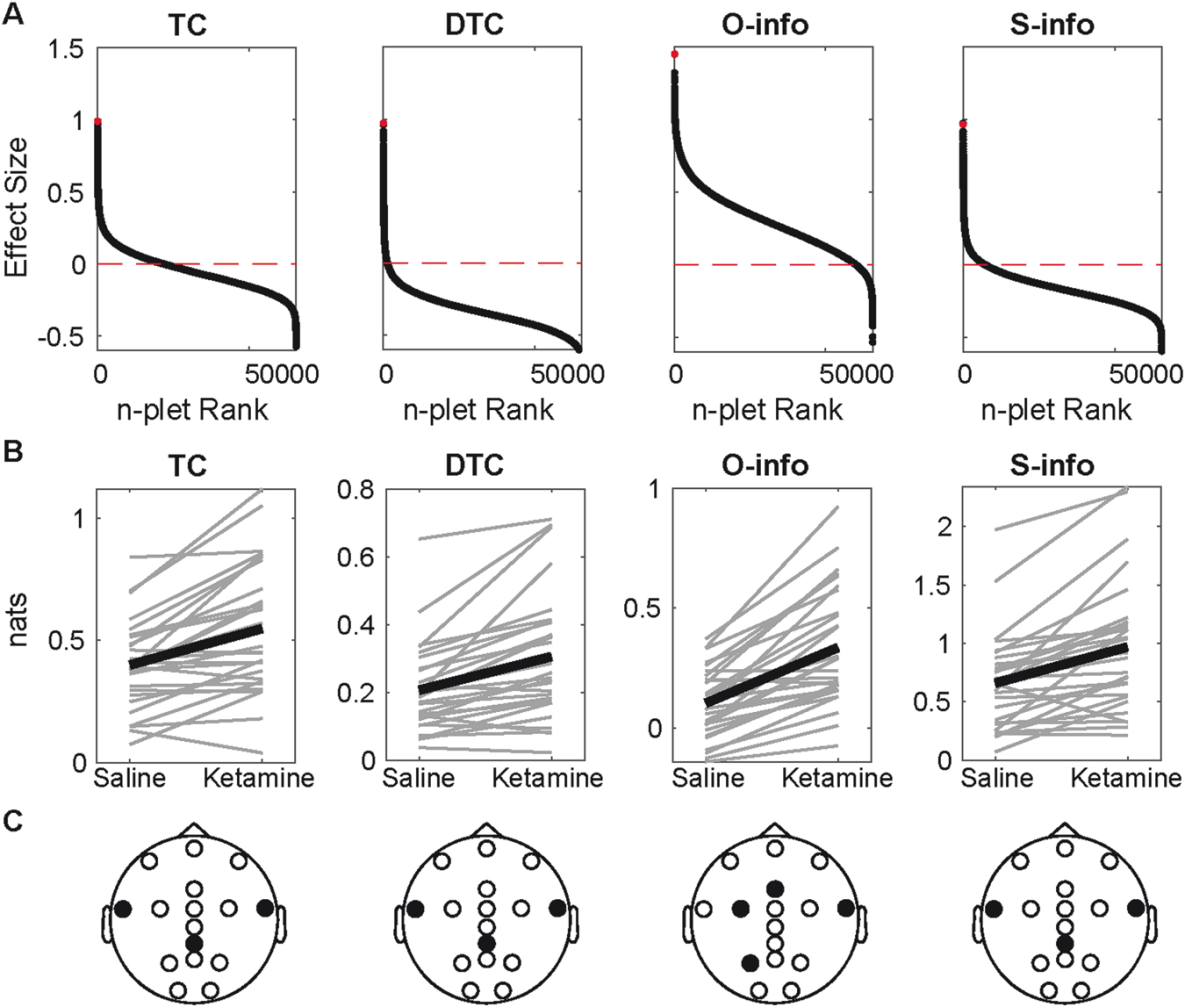
Ketamine increases redundancy in the resting state alpha band. **A** Each panel shows the effect sizes of each n-plet (i.e. each possible combination of electrodes from 2 to 16) sorted in decreasing order, with a red horizontal line showing the 0. The red dot denotes the maximum effect size. **B** The n-plet with the largest effect under saline and ketamine for each subject (gray) and for the average (black). Only O and S-info yielded FDR-corrected Wilcoxon sign rank p-values < 0.001. **C** EEG layout with the electrodes composing the n-plet in black.

Results evidenced both positive and negative effect sizes for all the combinations between filtering bands and metrics. The alpha band showed the largest (metric increase with ketamine) and smallest effect sizes (metric decrease with ketamine) for all the metrics (Fig. 2A and S1). Indeed, only O-info and S-info significantly increased in the alpha band (Fig. 1B, Wilcoxon sign rank test, p < 0.001 after FDR correction). Among the 4 metrics in the alpha band, the largest increase was found for the O-info, which included the P3, FCC3, Fz, and FT8 electrodes (Fig. 2C). The S-info was related to the CPz, FT7, and FT8 electrodes. Despite the other filtering bands also exhibiting a marked tendency both for increases and decreases, no significant effect was found (Fig. S2). These results indicate that the effect of ketamine can be better explained as an increase in the overarching correlations—specifically redundancy—between temporal and parietal recordings in the alpha band during the resting state.

To assess if EEG dynamics revealed through HOI analysis were also reflected by conventional pairwise connectivity analyses, pairwise connectivity was investigated using information theory metrics, specifically mutual information and conditional mutual information. The ability of a machine-learning classifier informed with EEG pairwise functional connectivity to discriminate conscious states was assessed, and connections best explaining the classification model were identified. The classification power of the model was low (AUC < 0.8), and connections contributing most to the classification model did not differ between states of consciousness (Wilcoxon sign rank test, p > 0.1) (Fig. S5). These results underscore the validity of HOI analysis to effectively capture EEG dynamics that are not observable through pairwise analyses.

### Ketamine increases redundancy for predictable stimuli

**Fig. 3:**
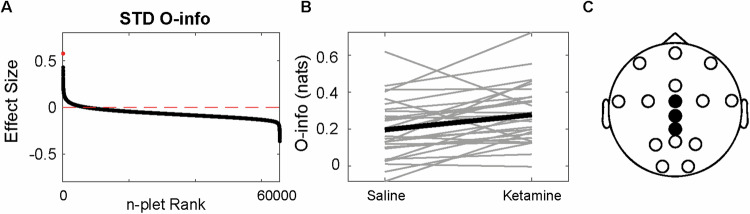
Redundancy increases in the standard tone for mismatch negativity task. **A** Effect sizes of each n-plet for the oddball paradigm, sorted in decreasing order, with a red horizontal line showing the 0. The red dot denotes the maximum effect size **B** The n-plet with the largest effect under saline and ketamine for each subject (gray) and for the average (black). **C** The electrodes involved in the best feature. Wilcoxon sign rank FDR-corrected p-value < 0.005.

We found both positive and negative effect sizes for all the combinations between metrics and stimuli type (STD, standard; DEV, deviant) for the auditory oddball task (Figs. 3A and S4). We compared the STD and DEV response under saline and under ketamine, without comparing the STD to the DEV tone. The deviant tone showed slightly larger absolute effect sizes. Significant differences were found only for the O-info in the standard tone (Fig. 3B, FDR-corrected Wilcoxon sign rank test, p < 0.005), which included CPz, Cz and FCz electrodes. The results for the rest of the metrics and the deviant tones are shown in Fig. S4. The same set of electrodes was found for both conditions in the tasks. These results indicate that ketamine significantly increases the evoked responses for predictable but not deviant stimuli.

### Derealization correlates with changes in resting state theta-band high-order interactions

**Fig. 4:**
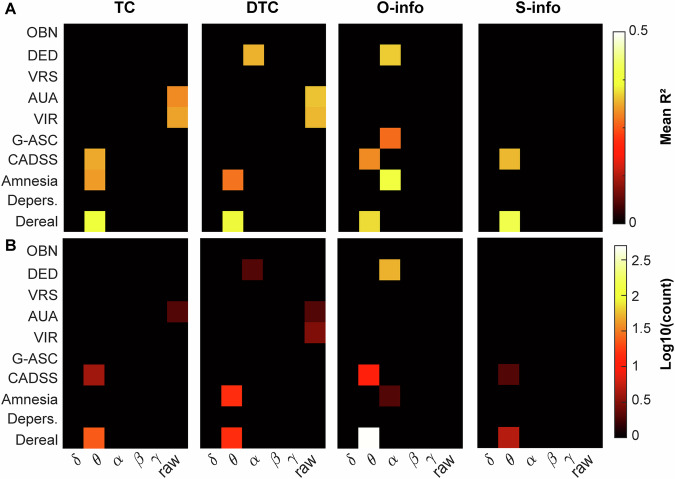
Association between subjective scores and HOI. **A** Average R2 only for the significant (FDR, p < 0.005) associations between the change in each dimension of subjective score (see section “Methods”) and change in HOI. **B** Same as **A**, but showing the number of significant HOI in log10 scale to improve visualization.

Finally, we investigated the association between the change in resting state HOI and the change in subjective scores, as measured via the CADSS and the 5D-ASC (see section “Methods”). We found the largest number of significant correlations for the O-info in the theta band and CADSS Derealization score, followed by the alpha band in the ‘Dread of ego dissolution’ item (Fig. 4B). Indeed, the theta band showed the largest association values for all the subjective scores, followed by the alpha band (Fig. S6). The presence of the alpha band is consistent with results of the previous section, where the largest effect sizes were found in the alpha-band.

This finding suggests that changes in the alpha band are not only indicative of the change in the global state of consciousness, but also of more subtler aspects of the experience. In turn, changes in the theta band HOI may be indicative of the level of derealization experienced by the subjects following ketamine infusion, as compared to saline.

## Discussion

This study aimed to investigate the neuropharmacological effects of ketamine on consciousness, employing a novel and robust approach using HOI, with portable EEG in a double-blinded cross-over design. This exploration centered around low-density arrays, addressing a current gap in research methodology. Key findings indicate that ketamine administration induced a significant increase in the correlations and redundancy of brain dynamics, particularly evident in the alpha frequency band, consistent with observations across classical EEG studies of ketamine. Results open a new avenue for future studies using portable, low-density recordings captured during pharmacological interventions that maximize the combined information across electrodes.

Our findings bolster the application of HOI for low-density arrays. The shifts in brain dynamics, specifically an increase in redundancy, are most pronounced in alpha (echoing previous reports focused on this band, tasks and rest [52–54]). Such alterations, particularly in the resting state, supports the potential of ketamine to decrease top-down control and increase the sensitization to bottom-up signals [1, 2, 12–15]. This phenomenon is manifested by an upswing in redundancy within brain dynamics, possibly reflecting a reduced top-down inhibition and an amplification of lower hierarchies of sensory information [12–15]. The increase in redundancy following ketamine administration was larger during resting states than tasks, also suggesting the spontaneous shift of the conscious state towards less controlled states during resting state, as dissociation. Also, the app-based auditory oddball paradigm revealed that the effects on redundancy are more conspicuous for predictable (standard) stimuli than for deviant stimuli, accentuating ketamine’s influence on spontaneous, less controlled cognitive processes [12, 15], as low-level stimuli prediction. Furthermore, a significant correlation emerged between the effects of ketamine on resting-state HOI in the alpha and theta band and the subjective experiences of DED and derealization, respectively, both core facets of ketamine-induced dissociative states. DED refers to a change in the perception of selfhood and subject-object boundaries, while derealization is the feeling of disconnection from the surroundings [12, 15, 16]. These results confirm reported associations between the alpha-band and ´*ego-integrity’* [55] as well as derealization measures and theta band [56, 57], particularly observed with ketamine [12]. Although it has been reported that ketamine modulates the gamma band [46, 58], our results show that changes in power spectrum can be dissociated from changes in HOI (we confirmed the gamma modulation in Figure S1), suggesting that these two aspects of brain dynamics could reflect different processes. Our findings support the idea that subjective alterations in consciousness are anchored in changes of brain dynamics and functional organization, and thus can be properly tracked with measures of neural complexity [59].

This study has limitations that open different avenues for further research. Compared to high-density electrodes, low-density dry electrodes offer limited information and have a lower signal-to-noise ratio. However, we attempted to mitigate these shortcomings with several internal controls in our design, including (a) no significant difference in the number of artifacts across conditions; (b) using a robust double-blinded cross-over design, which minimized individual heterogeneity, variance, and the distribution of random effects from any confounding variables across conditions; (c) verification of the expected effects of ketamine on the power spectrum (supplementary data). Indeed, the investigation of the minimal electrode layout that maximizes information retrieval from brain signals can potentially make EEG more accessible for scientific and clinical application but this is out of the scope of our current work. Further, we found a systematic directional consistency of the effects of HOI across various measures. Moreover, since the primary HOI differentiating the ketamine vs. saline conditions did not involve frontal areas, the results reduce the possibility that differences in eye movement across conditions could account for these effects. Despite these measures, our findings require further validation using high-density electrode arrays, though this might entail reducing the number of HOI. Exploring other brain measures like fMRI, where HOI can be assessed, would also be beneficial. While our sample size was modest, it remains comparable to, or even larger than, similar studies in the field (see reviews: [5, 6, 12, 13]). In any case, future studies need to replicate the findings in larger samples. This study only included male participants, so the effects of gender and the potential particular changes observed in women require further investigation. Lastly, our study lacks an exploration of brain dynamics in correlation with plasma measurements of ketamine and potential whole-body effects, such as cardio-dynamics and sensorimotor activity. These aspects offer promising avenues for future research.

## Conclusions

Our results suggest that HOI provide a novel approach to maximizing the information obtained from low-density EEG in pharmacological interventions. More specifically, ketamine fosters an increase in redundancy and HOI across measures suggesting changes in the way the brain processes information, leading towards a state of dissociation. Further research is needed to evaluate the potential of HOI to track the potential therapeutic effect of ketamine for psychiatric diseases such as depression. These effects offer a deeper understanding of the neuropharmacological actions of ketamine and underscore the potential of portable EEG devices in charting alterations in consciousness, especially when combined with the HOI in low-density setups. This could lay the foundation for future endeavors aimed at better capturing the subject’s preparedness and the subsequent pharmacological-induced changes in therapeutic settings across neuropsychiatric conditions. Finally, the use of portable EEG to track the effect of psychopharmacological interventions in non-clinical environments could enhance the accessibility to neurological exams especially for patients with reduced mobility.

### Supplementary information

Supplementary Material

## Data Availability

Computational codes written in MATLAB are available upon request to the first author, however, for preserving privacy, EEG data cannot be provided. Only data strictly necessary to reproduce the figures can be shared upon request.

## References

[1] Ballard ED, Zarate CA Jr. The role of dissociation in ketamine’s antidepressant effects. Nat Commun. 2020;11:6431. 33353946 10.1038/s41467-020-20190-4 PMC7755908

[2] Krystal JH, Karper LP, Seibyl JP, Freeman GK, Delaney R, Douglas Bremner J, et al. Subanesthetic effects of the noncompetitive NMDA antagonist, ketamine, in humans: psychotomimetic, perceptual, cognitive, and neuroendocrine responses. Arch Gen Psychiatry. 1994;51:199–214. 8122957 10.1001/archpsyc.1994.03950030035004

[3] Murrough JW, Abdallah CG, Mathew SJ. Targeting glutamate signalling in depression: progress and prospects. Nat Rev Drug Discov. 2017;16:472–86. 28303025 10.1038/nrd.2017.16

[4] Caddy C, Giaroli G, White TP, Shergill SS, Tracy DK. Ketamine as the prototype glutamatergic antidepressant: pharmacodynamic actions, and a systematic review and meta-analysis of efficacy. Ther Adv Psychopharmacol. 2014;4:75–99. 24688759 10.1177/2045125313507739 PMC3952483

[5] Schwertner A, Zortea M, Torres FV, Caumo W. Effects of subanesthetic ketamine administration on visual and auditory event-related potentials (ERP) in humans: a systematic review. Front Behav Neurosci. 2018;12:70. 29713269 10.3389/fnbeh.2018.00070 PMC5911464

[6] Zavaliangos-Petropulu A, Al-Sharif NB, Taraku B, Leaver AM, Sahib AK, Espinoza RT, et al. Neuroimaging-derived biomarkers of the antidepressant effects of ketamine. Biol Psychiatry Cogn Neurosci Neuroimaging. 2023;8:361–86. 36775711 10.1016/j.bpsc.2022.11.005 PMC11483103

[7] Schartner MM, Carhart-Harris RL, Barrett AB, Seth AK, Muthukumaraswamy SD. Increased spontaneous MEG signal diversity for psychoactive doses of ketamine, LSD and psilocybin. Sci Rep. 2017;7:46421. 28422113 10.1038/srep46421 PMC5396066

[8] Vlisides PE, Bel-Bahar T, Nelson A, Chilton K, Smith E, Janke E, et al. Subanaesthetic ketamine and altered states of consciousness in humans. Br J Anaesth. 2018;121:249–59. 29935579 10.1016/j.bja.2018.03.011 PMC6200112

[9] Bonhomme V, Vanhaudenhuyse A, Demertzi A, Bruno M-A, Jaquet O, Bahri MA, et al. Resting-state network-specific breakdown of functional connectivity during ketamine alteration of consciousness in volunteers. Anesthesiology. 2016;125:873–88. 27496657 10.1097/ALN.0000000000001275

[10] Zacharias N, Musso F, Müller F, Lammers F, Saleh A, London M, et al. Ketamine effects on default mode network activity and vigilance: A randomized, placebo-controlled crossover simultaneous fMRI/EEG study. Hum Brain Mapp. 2020;41:107–19. 31532029 10.1002/hbm.24791 PMC7268043

[11] Carhart-Harris RL, Leech R, Hellyer PJ, Shanahan M, Feilding A, Tagliazucchi E, et al. The entropic brain: a theory of conscious states informed by neuroimaging research with psychedelic drugs. Front Hum Neurosci. 2014;8:20. 24550805 10.3389/fnhum.2014.00020 PMC3909994

[12] de la Salle S, Choueiry J, Shah D, Bowers H, McIntosh J, Ilivitsky V, et al. Effects of ketamine on resting-state EEG activity and their relationship to perceptual/dissociative symptoms in healthy humans. Front Pharmacol. 2016;7:348. 27729865 10.3389/fphar.2016.00348 PMC5037139

[13] Nugent AC, Ballard ED, Gould TD, Park LT, Moaddel R, Brutsche NE, et al. Ketamine has distinct electrophysiological and behavioral effects in depressed and healthy subjects. Mol Psychiatry. 2019;24:1040–52. 29487402 10.1038/s41380-018-0028-2 PMC6111001

[14] Yang S, Seo H, Wang M, Arnsten AFT. NMDAR neurotransmission needed for persistent neuronal firing: potential roles in mental disorders. Front Psychiatry. 2021;12:654322. 33897503 10.3389/fpsyt.2021.654322 PMC8064413

[15] Carhart-Harris RL, Friston KJ. REBUS and the anarchic brain: toward a unified model of the brain action of psychedelics. Pharmacol Rev. 2019;71:316–44. 31221820 10.1124/pr.118.017160 PMC6588209

[16] Rajpal H, Mediano PAM, Rosas FE, Timmermann CB, Brugger S, Muthukumaraswamy S, et al. Psychedelics and schizophrenia: distinct alterations to Bayesian inference. Neuroimage. 2022;263:119624. 36108798 10.1016/j.neuroimage.2022.119624 PMC7614773

[17] Sanz Perl Y, Bocaccio H, Pallavicini C, Pérez-Ipiña I, Laureys S, Laufs H, et al. Nonequilibrium brain dynamics as a signature of consciousness. Phys Rev E. 2021;104:014411. 34412335 10.1103/PhysRevE.104.014411

[18] Dasilva M, Camassa A, Navarro-Guzman A, Pazienti A, Perez-Mendez L, Zamora-López G, et al. Modulation of cortical slow oscillations and complexity across anesthesia levels. Neuroimage. 2021;224:117415. 33011419 10.1016/j.neuroimage.2020.117415

[19] Byrom B, McCarthy M, Schueler P, Muehlhausen W. Brain monitoring devices in neuroscience clinical research: the potential of remote monitoring using sensors, wearables, and mobile devices. Clin Pharmacol Ther. 2018;104:59–71. 29574776 10.1002/cpt.1077 PMC6032823

[20] Whelan R, Barbey FM, Cominetti MR, Gillan CM, Rosická AM. Developments in scalable strategies for detecting early markers of cognitive decline. Transl Psychiatry. 2022;12:473. 36351888 10.1038/s41398-022-02237-w PMC9645320

[21] Herzog R, Haghayegh S, Ibáñez A, Hu K. 0052 a novel biomarker of Alzheimer’s disease based on high-order interactions of low-density electroencephalography. Sleep. 2023;46:A25–A25. 10.1093/sleep/zsad077.0052

[22] Battiston F, Amico E, Barrat A, Bianconi G, Ferraz de Arruda G, Franceschiello B, et al. The physics of higher-order interactions in complex systems. Nat Phys. 2021;17:1093–8. 10.1038/s41567-021-01371-4

[23] Zhu H, Wang J, Zhao Y-P, Lu M, Shi J. Contrastive multi-view composite graph convolutional networks based on contribution learning for autism spectrum disorder classification. IEEE Trans Biomed Eng. 2023;70:1943–54. 37015677 10.1109/TBME.2022.3232104

[24] Plis SM, Sui J, Lane T, Roy S, Clark VP, Potluru VK, et al. High-order interactions observed in multi-task intrinsic networks are dominant indicators of aberrant brain function in schizophrenia. Neuroimage. 2014;102:35–48. 23876245 10.1016/j.neuroimage.2013.07.041 PMC3896503

[25] Herzog R, Rosas FE, Whelan R, Fittipaldi S, Santamaria-Garcia H, Cruzat J, et al. Genuine high-order interactions in brain networks and neurodegeneration. Neurobiol Dis. 2022;175:105918. 36375407 10.1016/j.nbd.2022.105918 PMC11195446

[26] Varley TF, Pope M, Puxeddu MG, Faskowitz J, Sporns O. Partial entropy decomposition reveals higher-order information structures in human brain activity. Proc Natl Acad Sci USA. 2023;120:e2300888120. 37467265 10.1073/pnas.2300888120 PMC10372615

[27] Varley TF, Pope M, Faskowitz J, Sporns O. Multivariate information theory uncovers synergistic subsystems of the human cerebral cortex. Commun Biol. 2023;6:451. 37095282 10.1038/s42003-023-04843-w PMC10125999

[28] Frohlich J, Chiang JN, Mediano PAM, Nespeca M, Saravanapandian V, Toker D, et al. Neural complexity is a common denominator of human consciousness across diverse regimes of cortical dynamics. Commun Biol. 2022;5:1374. 36522453 10.1038/s42003-022-04331-7 PMC9755290

[29] Mediano PAM, Rosas FE, Bor D, Seth AK, Barrett AB. The strength of weak integrated information theory. Trends Cogn Sci. 2022;26:646–55. 35659757 10.1016/j.tics.2022.04.008

[30] Atasoy S, Deco G, Kringelbach ML, Pearson J. Harmonic brain modes: a unifying framework for linking space and time in brain dynamics. Neuroscientist. 2018;24:277–93. 28863720 10.1177/1073858417728032

[31] Mashour GA, Hudetz AG. Neural correlates of unconsciousness in large-scale brain networks. Trends Neurosci. 2018;41:150–60. 29409683 10.1016/j.tins.2018.01.003 PMC5835202

[32] Cofré R, Herzog R, Mediano PAM, Piccinini J, Rosas FE, Sanz Perl Y, et al. Whole-brain models to explore altered states of consciousness from the bottom up. Brain Sci. 2020;10. 10.3390/brainsci10090626. 10.3390/brainsci10090626 PMC7565030 32927678

[33] Muthukumaraswamy SD, Shaw AD, Jackson LE, Hall J, Moran R, Saxena N. Evidence that subanesthetic doses of ketamine cause sustained disruptions of NMDA and AMPA-mediated frontoparietal connectivity in humans. J Neurosci. 2015;35:11694–706. 26290246 10.1523/JNEUROSCI.0903-15.2015 PMC4540803

[34] Thiebes S, Steinmann S, Curic S, Polomac N, Andreou C, Eichler I-C, et al. Alterations in interhemispheric gamma-band connectivity are related to the emergence of auditory verbal hallucinations in healthy subjects during NMDA-receptor blockade. Neuropsychopharmacology. 2018;43:1608–15. 29453445 10.1038/s41386-018-0014-z PMC5983549

[35] McWilliams EC, Barbey FM, Dyer JF, Islam MN, McGuinness B, Murphy B, et al. Feasibility of repeated assessment of cognitive function in older adults using a wireless, mobile, dry-EEG headset and tablet-based games. Front Psychiatry. 2021;12:574482. 34276428 10.3389/fpsyt.2021.574482 PMC8281974

[36] Rosas FE, Mediano PAM, Gastpar M, Jensen HJ. Quantifying high-order interdependencies via multivariate extensions of the mutual information. Phys Rev E. 2019;100:32305. 10.1103/PhysRevE.100.032305 31640038

[37] Farnes N, Juel BE, Nilsen AS, Romundstad LG, Storm JF. Increased signal diversity/complexity of spontaneous EEG, but not evoked EEG responses, in ketamine-induced psychedelic state in humans. PLoS ONE. 2020;15:e0242056. 33226992 10.1371/journal.pone.0242056 PMC7682856

[38] Bredlau AL, Thakur R, Korones DN, Dworkin RH. Ketamine for pain in adults and children with cancer: a systematic review and synthesis of the literature. Pain Med. 2013;14:1505–17. 23915253 10.1111/pme.12182

[39] Lineham A, Avila-Quintero VJ, Bloch MH, Dwyer J. The relationship between acute dissociative effects induced by ketamine and treatment response in adolescent patients with treatment-resistant depression. J Child Adolesc Psychopharmacol. 2023;33:20–26. 36799961 10.1089/cap.2022.0086

[40] Acevedo-Diaz EE, Cavanaugh GW, Greenstein D, Kraus C, Kadriu B, Park L, et al. Can ‘floating’ predict treatment response to ketamine? Data from three randomized trials of individuals with treatment-resistant depression. J Psychiatr Res. 2020;130:280–5. 32861983 10.1016/j.jpsychires.2020.06.012 PMC8073211

[41] Barbey FM, Farina FR, Buick AR, Danyeli L, Dyer JF, Islam MN, et al. Neuroscience from the comfort of your home: Repeated, self-administered wireless dry EEG measures brain function with high fidelity. Front Digit Health. 2022;4:944753. 35966140 10.3389/fdgth.2022.944753 PMC9372279

[42] Duncan CC, Barry RJ, Connolly JF, Fischer C, Michie PT, Näätänen R, et al. Event-related potentials in clinical research: guidelines for eliciting, recording, and quantifying mismatch negativity, P300, and N400. Clin Neurophysiol. 2009;120:1883–908. 19796989 10.1016/j.clinph.2009.07.045

[43] Bremner JD, Krystal JH, Putnam FW, Southwick SM, Marmar C, Charney DS, et al. Measurement of dissociative states with the Clinician-Administered Dissociative States Scale (CADSS). J Trauma Stress. 1998;11:125–36. 9479681 10.1023/A:1024465317902

[44] Studerus E, Gamma A, Vollenweider FX. Psychometric evaluation of the altered states of consciousness rating scale (OAV). PLoS ONE. 2010;5:e12412. 20824211 10.1371/journal.pone.0012412 PMC2930851

[45] Nottage JF, Horder J. State-of-the-art analysis of high-frequency (Gamma Range) electroencephalography in humans. Neuropsychobiology. 2015;72:219–28. 26900860 10.1159/000382023

[46] Nottage JF, Gabay A, De Meyer K, Herrik KF, Bastlund JF, Christensen SR, et al. The effect of ketamine and D-cycloserine on the high frequency resting EEG spectrum in humans. Psychopharmacology. 2023;240:59–75. 36401646 10.1007/s00213-022-06272-9 PMC9816261

[47] Ince RAA, Giordano BL, Kayser C, Rousselet GA, Gross J, Schyns PG. A statistical framework for neuroimaging data analysis based on mutual information estimated via a gaussian copula. Hum Brain Mapp. 2017;38:1541–73. 27860095 10.1002/hbm.23471 PMC5324576

[48] Gatica M, Cofré R, Mediano PAM, Rosas FE, Orio P, Diez I, et al. High-order interdependencies in the aging brain. Brain Connect. 2021;11:734–44. 33858199 10.1089/brain.2020.0982

[49] Rosas FE, Mediano PAM, Luppi AI, Varley TF, Lizier JT, Stramaglia S, et al. Disentangling high-order mechanisms and high-order behaviours in complex systems. Nat Phys. 2022;18:476–7. 10.1038/s41567-022-01548-5

[50] Sawilowsky SS. New effect size rules of thumb. J Mod Appl Stat Methods. 2009;8:26. 10.22237/jmasm/1257035100

[51] Kriegeskorte N, Simmons WK, Bellgowan PSF, Baker CI. Circular analysis in systems neuroscience: the dangers of double dipping. Nat Neurosci. 2009;12:535–40. 19396166 10.1038/nn.2303 PMC2841687

[52] Anijärv TE, Can AT, Gallay CC, Forsyth GA, Dutton M, Mitchell JS, et al. Spectral changes of EEG following a 6-week low-dose oral ketamine treatment in adults with major depressive disorder and chronic suicidality. Int J Neuropsychopharmacol. 2023;26:259–67. 36789509 10.1093/ijnp/pyad006 PMC10109122

[53] Liley DTJ, Muthukumaraswamy SD. Evidence that alpha blocking is due to increases in system-level oscillatory damping not neuronal population desynchronisation. Neuroimage. 2020;208:116408. 31790751 10.1016/j.neuroimage.2019.116408

[54] Keavy D, Bristow LJ, Sivarao DV, Batchelder M, King D, Thangathirupathy S, et al. The qEEG signature of selective NMDA NR2B negative allosteric modulators; a potential translational biomarker for drug development. PLoS ONE. 2016;11:e0152729. 27035340 10.1371/journal.pone.0152729 PMC4817977

[55] Stoliker D, Egan GF, Friston KJ, Razi A. Neural mechanisms and psychology of psychedelic ego dissolution. Pharmacol Rev. 2022;74:876–917. 36786290 10.1124/pharmrev.121.000508

[56] Soffer-Dudek N, Todder D, Shelef L, Deutsch I, Gordon S. A neural correlate for common trait dissociation: Decreased EEG connectivity is related to dissociative absorption. J Pers. 2019;87:295–309. 29626343 10.1111/jopy.12391

[57] Krüger C, Bartel P, Fletcher L. Dissociative mental states are canonically associated with decreased temporal theta activity on spectral analysis of EEG. J Trauma Dissociation. 2013;14:473–91. 23796176 10.1080/15299732.2013.769480

[58] Lazarewicz MT, Ehrlichman RS, Maxwell CR, Gandal MJ, Finkel LH, Siegel SJ. Ketamine modulates theta and gamma oscillations. J Cogn Neurosci. 2010;22:1452–64. 19583475 10.1162/jocn.2009.21305

[59] Sarasso S, Casali AG, Casarotto S, Rosanova M, Sinigaglia C, Massimini M. Consciousness and complexity: a consilience of evidence. Neurosci Conscious. 2021;2021:niab023. 38496724 10.1093/nc/niab023 PMC10941977

