# Supplementary material for: High-order brain interactions in ketamine during rest and task: a double-blinded cross-over design using portable EEG on male participants
Source: Transl Psychiatry. 2024 Jul 27;14:310. doi: 10.1038/s41398-024-03029-0 (PMC11283531; doi:10.1038/s41398-024-03029-0)
Supplement: Supplementary file 1 — Supplementary Material [file 41398_2024_3029_MOESM1_ESM.pdf]

Supplementary Material for

High-order brain interactions in ketamine during rest and task:

A double-blinded cross-over design using portable EEG on male participants

Rubén Herzog [1,2], Florentine Marie Barbey[3], Md Nurul Islam[3], Laura Rueda-Delgado[3], Hugh Nolan[3], Pavel Prado[4], Marina Krylova[5], Igor Izyurov[5], Nooshin Javaheripour[5], Lena Vera Danyeli[5], Zümrüt Duygu Sen[5,], Martin Walter[5,6], Patricio O'Donnell[7], Derek L. Buhl[7], Brian Murphy[3], Agustin Ibanez[1,8,*]

1. Latin American Brain Health Institute, Universidad Adolfo Ibañez, Santiago de Chile, Chile

2. Sorbonne Université, Institut du Cerveau - Paris Brain Institute - ICM, Inserm, CNRS, Paris, France

3. Cumulus Neuroscience Ltd, Dublin, Ireland.

4. Escuela de Fonoaudiología, Facultad de Odontología y Ciencias de la Rehabilitación, Universidad San Sebastián, Santiago, Chile

5. Department of Psychiatry and Psychotherapy, Jena University Hospital, Jena, Germany

6. German Center for Mental Health (DZPG), partner site Halle-Jena-Magdeburg, Germany

7. Neuroscience Drug Discovery Unit, Takeda Pharmaceuticals, 350 Massachusetts Avenue, Cambridge, MA 02390, USA

8. Global Brain Health Institute, UCSF and Trinity College Dublin.

* Corresponding author: A. Ibanez (agustin.ibanez@gbhi.org) and R. Herzog (rherzoga@gmail.com)

1 Supplementary section

2 Supplementary tables

Supplementary table 1. Resting state data used for the analysis plus statistical analysis of the number of selected points. No significant difference was found between the selected population.

sub/[points]	Saline	Ket	status
'1502'	144901	146242	
'1503'	146109	142900	
'1504'	141626	146372	
'1505'	141639	145780	
'1506'	146363	146118	
'1507'	146217	141439	
'1508'	142087	146599	
'1509'	142805	146220	
'1510'	133951	137001	
'1511'	146411	138588	
'1512'	144914	144817	
'1513'	143412	372	excluded
'1516'	146539	126010	
'1517'	143007	129549	
'1518'	142667	146207	
'1521'	137691	146445	
'1522'	99789	143479	
'1523'	143149	130973	
'1525'	143697	145009	
'1527'	135146	104935	
'1528'	123987	73125	
'1530'	146119	146251	
'1531'	138838	146377	
'1534'	146387	138865	
'1537'	142611	146385	
'1539'	143851	137336	
'1540'	141464	144438	
'1541'	145920	146079	
'1543'	133610	138451	
Mean	140514,0345	133529,7241	
SD	9311,737081	29852,41772	
Max	146539	146599	
Min	99789	372	
Wilcoxon p-val		0,5814	

Supplementary Table 2. Auditory oddball task data used for the analysis plus statistical analysis of the number of selected points and trials. No significant difference was found between the selected population.

STD	[points]			DEV	[points]			STD	Trials			STD	Trials	
sub	Saline	Ket			Saline	Ket			Saline	Ket			Saline	Ket
'1502'	184728	108256			36852	21551			986	599			197	119
'1503'	187309	187321			37305	37327			1000	1000			199	199
'1504'	184927	186034			36873	37007			988	992			197	198
'1505'	177340	162964			35386	33013			951	879			190	180
'1506'	185188	187601			36611	37285			988	1000			196	199
'1507'	182735	184732			36154	36936			977	987			193	197
'1508'	181592	157361			36035	31733			971	844			193	170
'1509'	181442	185905			35877	37036			972	993			192	198
'1510'	122361	151999			24082	30591			668	828			131	165
'1511'	187668	170277			37157	33936			1000	913			199	182
'1512'	180822	142635			36244	28733			967	776			194	157
'1513'	176199	176084			35317	35038			944	943			190	188
'1516'	187598	140265			37326	26995			1000	754			199	145
'1517'	185267	152223			37104	30436			989	826			198	166
'1518'	182690	180349			36145	36029			977	967			193	192
'1521'	176584	186257			35368	36840			945	993			190	197
'1522'	187434	182296			37297	36368			1000	978			199	195
'1523'	177551	186538			35188	37106			950	994			188	198
'1525'	183209	187654			36383	37234			980	1000			194	199
'1527'	121913	138603			24138	28020			667	757			131	154
'1528'	150135	137364			29985	27479			812	740			163	148
'1530'	186030	185356			36847	36382			993	989			197	196
'1531'	179732	177590			35696	35392			961	952			192	189
'1534'	185147	180725			36629	35749			987	969			197	191
'1537'	162506	187425			32532	37370			874	1000			175	199
'1539'	184919	182806			36714	36573			987	976			196	196
'1540'	180443	182116			35967	36415			965	973			192	195
'1541'	168346	186023			33709	37043			905	993			182	198
'1543'	167458	171731			33193	34093			897	922			178	183
Mean	175837	170568,6207			34969,44828	33990			941,4137931	915,0689655			187,4137931	182,5172414
SD	17079,8056	20788,46487			3415,982034	4105,624296			86,89094858	105,4540967			17,58757118	20,88680494
Max	187668	187654			37326	37370			1000	1000			199	199
Min	121913	108256			24082	21551			667	599			131	119
Wilcoxon p-val		0,5235				0,6114				0,5538				0,6479

Supplementary Table 3: Linear regression on the recorded tablet volume against the session number, that is the number of times a user has performed the MMN task. Neither initial volume or final volume - i.e. volume after incorporating any volume changes within the task - demonstrated an increase with session number.

	Coef.	STD error	t	P>|t|	[0.025	0.975]
Intercept	7.04	0.20	34.90	0.000	6.65	7.44

3 Supplementary figures

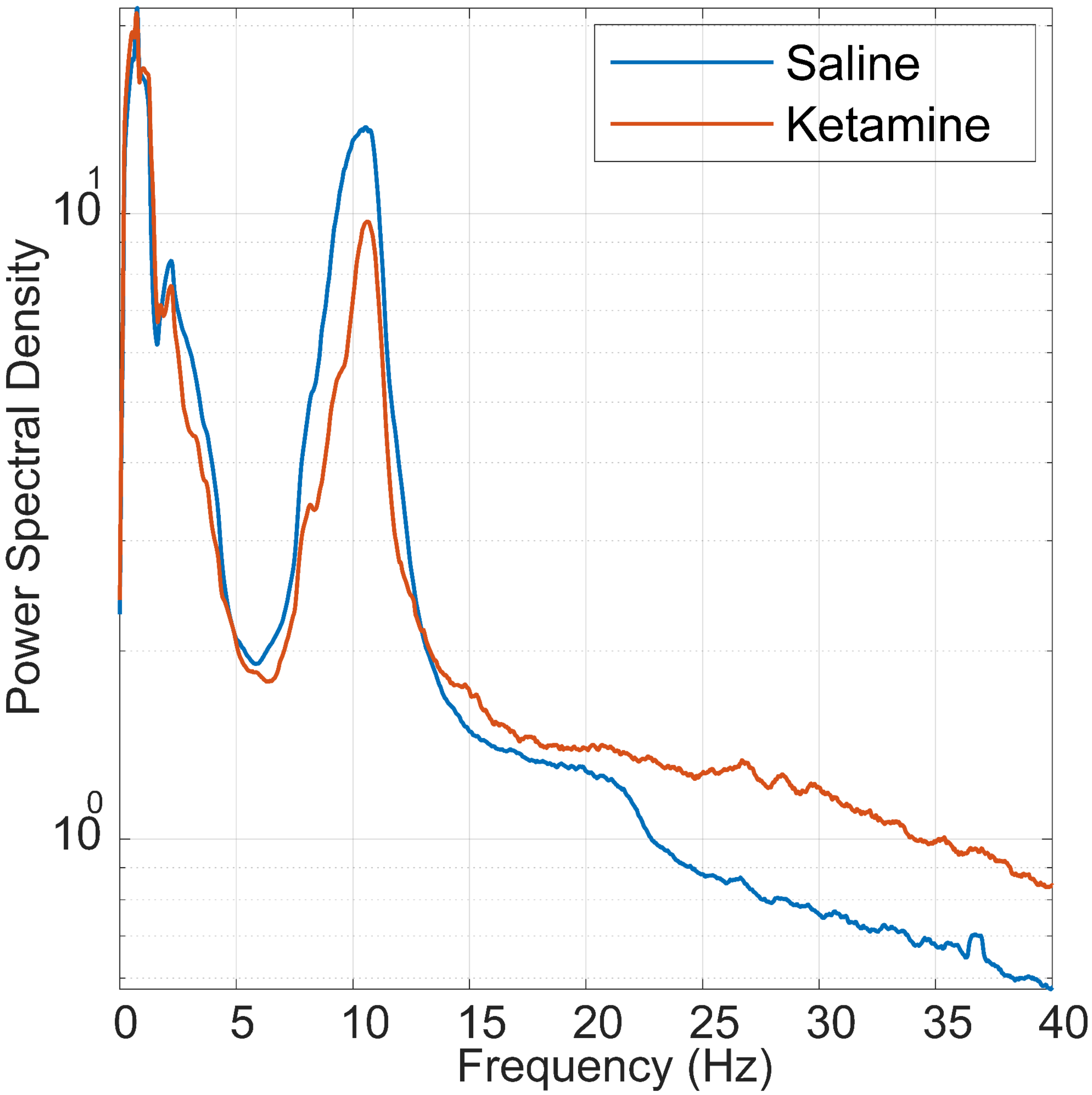

Figure S1. Average (over all electrodes and subjects) power spectral densities for saline and ketamine during resting state. Ketamine, as other psychedelic drugs, reduces the alpha peak and flattens the slope of the spectrum, consistent with previous reports (https://www.sciencedirect.com/science/article/pii/S0007091218302381).

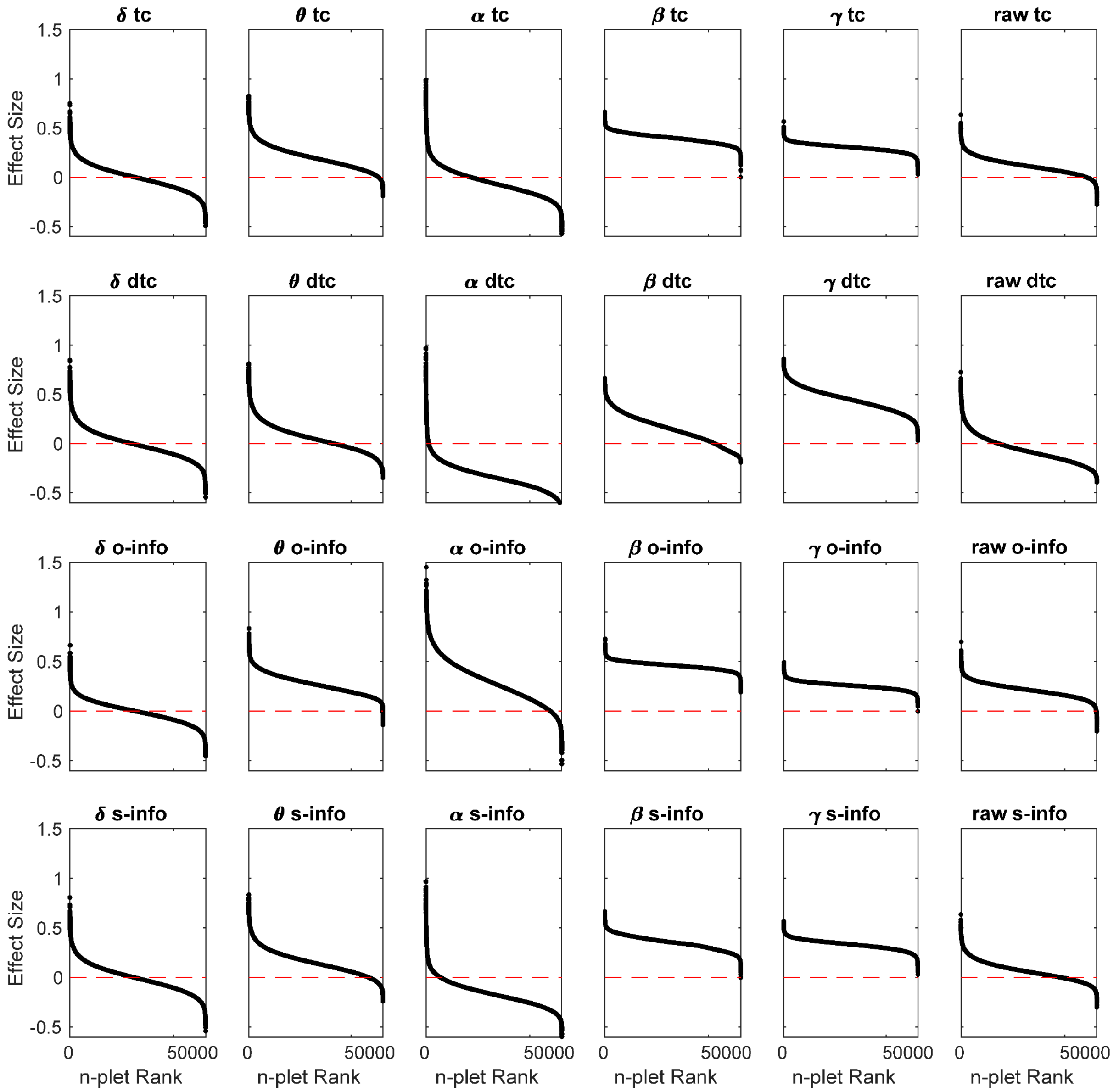

Figure S2. Effect sizes for resting state for all measures and filtering bands. Rows are the different metrics (TC, DTC, O-info and S-info) and columns are the different filtering bands. Each panel shows the effect sizes of each n-plet (i.e. each possible combination of electrodes from 2 to 16) sorted in decreasing order, with a red horizontal line showing the 0. Note that the alpha band shows the largest effect sizes.

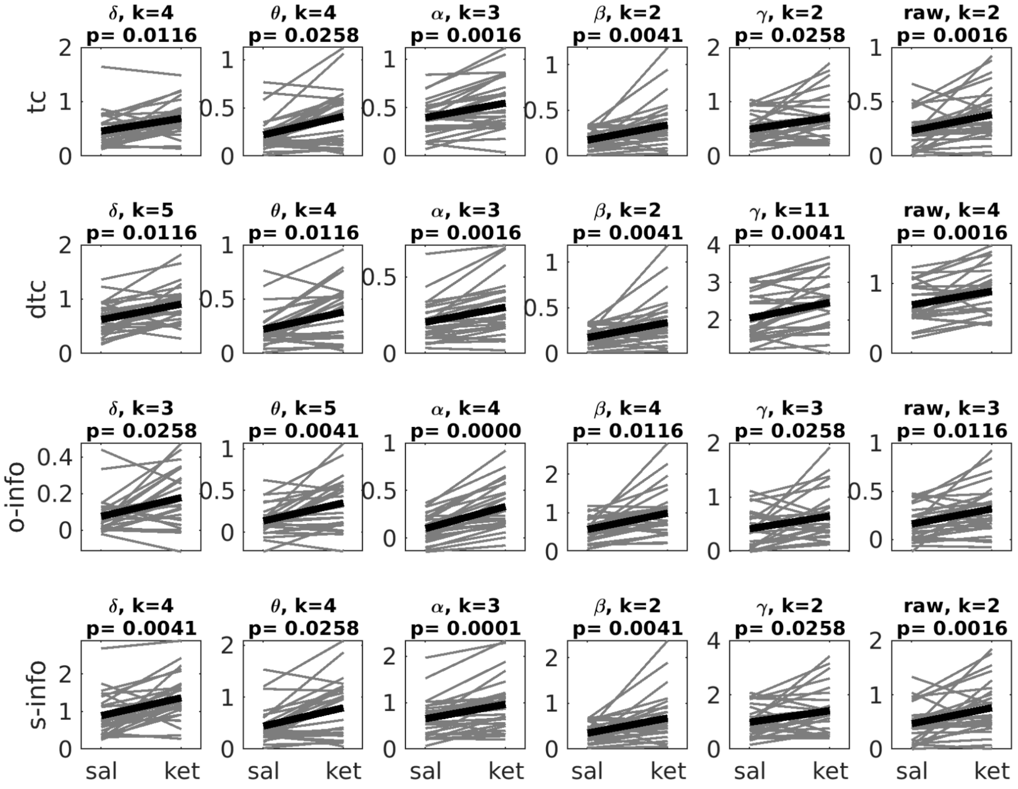

Figure S3. Best n-plet per measure and filtering band for resting state. Rows are the different metrics (TC, DTC, O-info and S-info) and columns are the different filtering bands. Each panel shows the value of the n-plet with the largest effect under saline (sal) and ketamine (ket) for each subject (gray) and for the average (black). k is the order of interactions of the corresponding n-plet and p the FDR corrected p-value. For the alpha band the O and S-information yield significant differences (Wilcoxon sign rank test, $p<0.001$) using high order interactions (more than 2 electrodes).

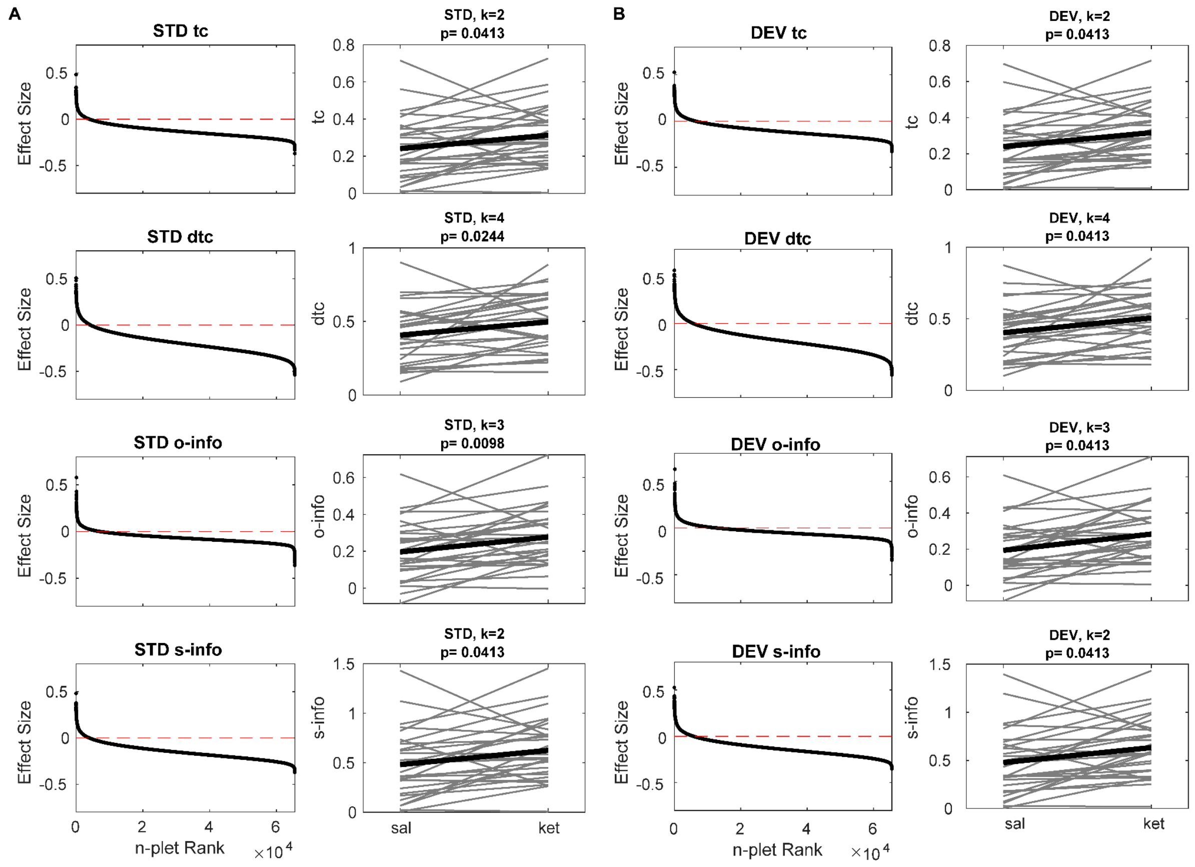

Figure S4. Effect sizes and best n-plets for auditory oddball task. A) Sorted effect sizes and best n-plet for each metric (rows) for the standard (STD) tone. k indicates the order of interactions and p the corresponding FDR corrected p-value. B) Same as A but for the deviant (DEV) tone. Only O-info yielded p-values<0.005 (FDR Wilcoxon sign rank test corrected).

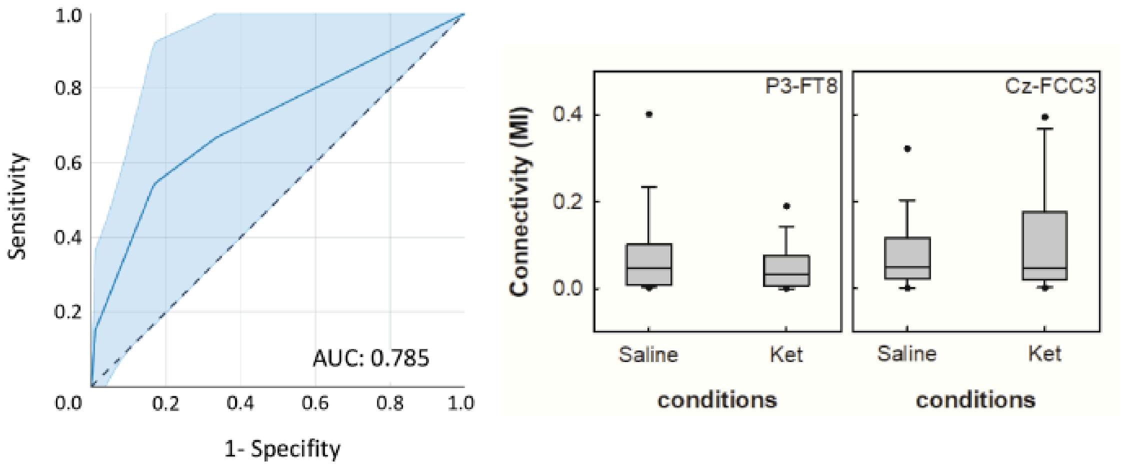

Figure S5. EEG pairwise connectivity analysis using theoretic information metrics. Saline and Ketamine (Ket) conditions were discriminated by a machine learning classifier informed with pairwise functional connections estimated using mutual information (MI) and conditional mutual information. Left panel illustrates the ROC curve. Right panel shows the connections most contributing to the model explanation. Top connections represented interactions between P3-FT8 and Cz-FCC8, both captured by MI. Connections did not have statistically significant differences between conditions as reflected by the Wilcoxon sign rank test for paired samples (p=0.22, and p=0.84 for P3-FT8 and Cz-FCC8, respectively). AUC: area under the curve

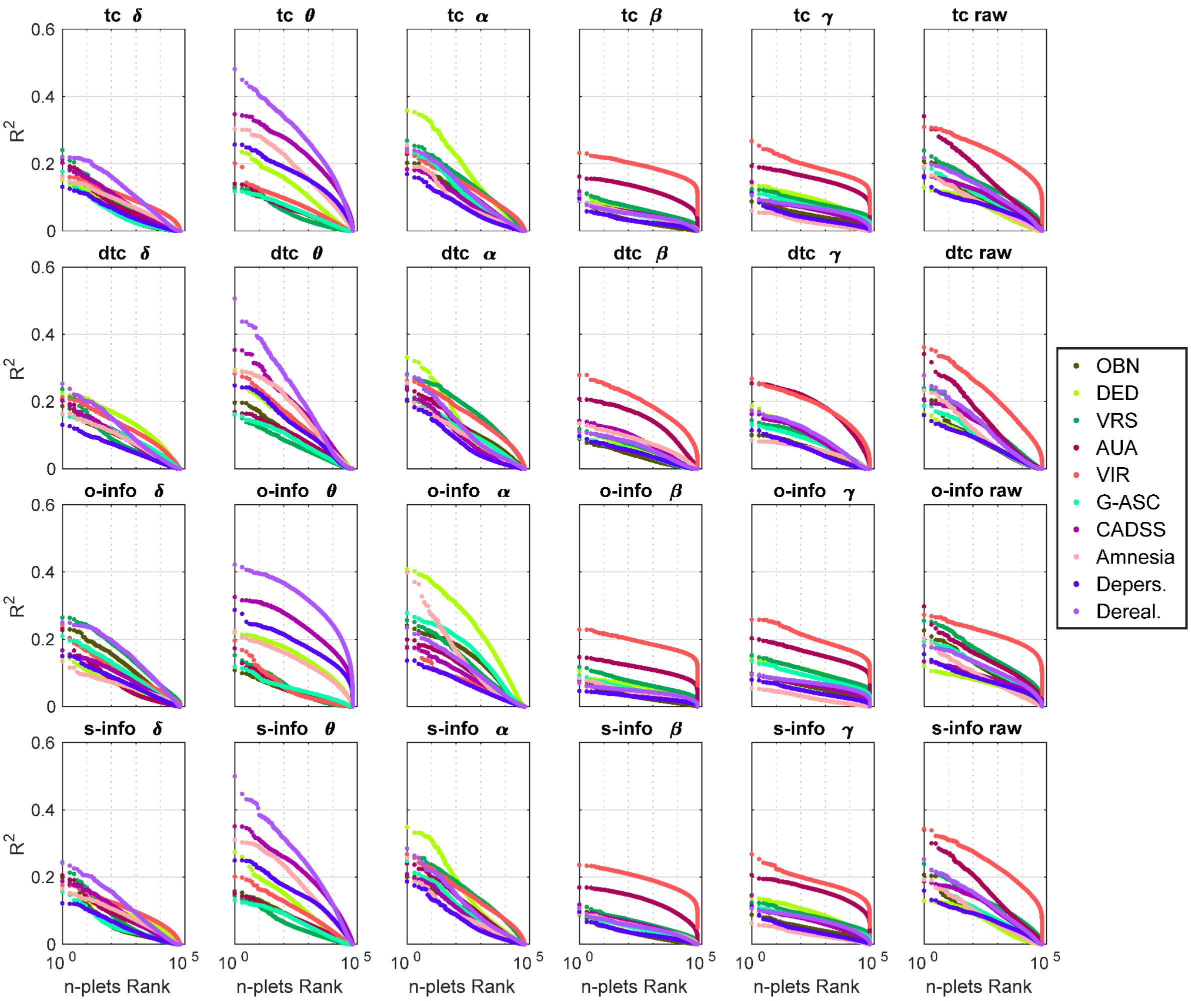

Figure S6. Association (R^2) between the change in HOI and the change in subjective scores. Each row is a metric and each column a different filtering band. Colors are subjective scores (see Methods). The x-axis is in logarithmic scale. Note that the theta band shows the largest values.
